# miR-19b downregulates intestinal SOCS3 to reduce intestinal inflammation in Crohn’s disease

**DOI:** 10.1038/srep10397

**Published:** 2015-05-22

**Authors:** Xiuqin Cheng, Xiaofei Zhang, Jiewen Su, Yingdi Zhang, Weimei Zhou, Jun Zhou, Cheng Wang, Hongwei Liang, Xi Chen, Ruihua Shi, Ke Zen, Chen-Yu Zhang, Hongjie Zhang

**Affiliations:** 1 Department of Gastroenterology, First Affiliated Hospital of Nanjing Medical University, Nanjing, Jiangsu 210029, China; 2 Jiangsu Engineering Research Center for MicroRNA Biology and Biotechnology, State Key Laboratory of Pharmaceutical Biotechnology, School of Life Sciences, Nanjing University, 22 Hankou Road, Nanjing, Jiangsu 210093, China; 3 Department of Gastroenterology, Laigang Hospital Affiliated to Taishan Medical University,Laiwu, Shandong 271000, China

## Abstract

Although aberrant microRNA (miRNA) expression has frequently been observed in inflammatory bowel disease (IBD), its biological functions and targets remain largely unknown. Present study found that miR-19b was significantly downregulated in active Crohn’s disease (CD). Using bioinformatics analysis, suppressor of cytokine signalling 3 (SOCS3), a physiological regulator of innate and adaptive immunity that controls several immuno-inflammatory diseases, was predicted to be a potential target of miR-19b. An inverse correlation between miR-19b and SOCS3 protein levels, but not mRNA, was identified in active-CD intestinal tissue samples. By overexpressing or knocking down miR-19b in Caco2 cells and HT29 cells, it was experimentally validated that miR-19b is a direct regulator of SOCS3. Using a luciferase reporter assay, it was confirmed that miR-19b directly recognizes the 3’-untranslated region (3’-UTR) of SOCS3. Furthermore, overexpression of miR-19b decreased SOCS3 expression, leading to increased production of macrophage-inflammatory protein-3α (MIP-3α) in Caco2 cells. In contrast, knockdown of miR-19b increased SOCS3 and decreased MIP-3α. Finally, intracolonically delivered miR-19b decreased the severity of colitis induced with 2,4,6-trinitrobenzene sulphonic acid (TNBS). Taken together, our findings suggest that miR-19b suppresses the inflammatory response by inhibiting SOCS3 to modulate chemokine production in intestinal epithelial cells (IECs) and thereby prevents the pathogenesis of CD.

Inflammatory bowel disease (IBD) is characterized by chronic recurring gastrointestinal inflammation, primarily classified into two major phenotypes: Crohn’s disease (CD) and ulcerative colitis (UC). The precise etiology of CD remains largely unknown, although current evidence suggests that CD is caused by complex interactions between environmental, genetic, and immuno-regulatory factors. In particular, immune dysregulation is thought to play a significant role in the pathogenesis of CD^1^,^2^.

Various cytokines are involved in innate and adaptive immune regulation, and dysregulation of cytokine signalling contributes to heightened inflammation and diseases such as autoimmune disease^3^. Cytokines often act through the Janus kinase/signal transducer and activator of transcription (JAK/STAT) pathway, which is negatively regulated by suppressors of cytokine signalling (SOCS) proteins^4^. SOCS proteins are key physiological regulators of innate and adaptive immunity and control immuno-inflammatory disease development^5^,^6^. The SOCS family includes eight proteins: SOCS1-SOCS7 and CIS, each of which contains a central Src-homology 2 (SH2) domain and a C-terminal SOCS box^7^. These proteins bind to JAK or cytokine receptors to suppress downstream signalling events^8^. Among the SOCS family members, SOCS1 and SOCS3 are key regulators of innate and adaptive immunity^6^,^9^.

Because SOCS3 regulates multiple cytokine signalling pathways, it may be a useful therapeutic target for autoimmune disease^9^,^10^. SOCS3 expression is increased in inflamed tissue compared to normal tissue, and its expression is particularly high in recruited leukocytes and the epithelium^11^. Intestinal epithelial cells (IECs) have been consistently linked to IBD pathogenesis. During the course of intestinal inflammation or microbial infection, IECs exhibit a classic inflammatory response in addition to their normal absorptive and secretory functions^12^,^13^. High SOCS3 expression has been observed in IBD, although the role of SOCS3 in IBD remains unclear^14^. It is important to understand what molecules regulate SOCS3 expression to identify potential therapeutic targets for anti-inflammatory therapies.

MicroRNAs (miRNAs) are non-coding RNA molecules (21–23 nucleotides in length) that post-transcriptionally regulate gene expression. miRNA binding to complementary sequences in the 3-untranslated region (UTR) of target mRNA molecules results in mRNA degradation or translational inhibition^15^. Although most miRNA target genes have not been identified, miRNAs have been implicated in several cellular processes, including differentiation, proliferation, maturation, and apoptosis. Furthermore, there is accumulating evidence that miRNAs regulate inflammatory processes^16^,^17^. Differential miRNA expression has been described in autoimmune diseases, suggesting that miRNA regulation could be involved in autoimmune disease development or prevention, including conditions such as psoriasis, rheumatoid arthritis (RA), and systemic lupus erythematosus^18^,^19^. Recently, unique miRNA expression profiles have been described in active UC and CD patient epithelia^17^,^20^. Our previous study showed that several miRNAs are differentially expressed in the intestine of active CD patients. However, differential expression of miRNAs and their roles in epithelial disruption during IBD remain unclear. In the current study, we hypothesized that intestinal epithelia disruption is linked to abnormal SOCS3 expression and that miRNAs regulate this abnormal expression during intestinal inflammation.

## Results

### SOCS3 protein, but not SOCS3 mRNA, is upregulated in active CD intestinal mucosa

**Figure 1:**
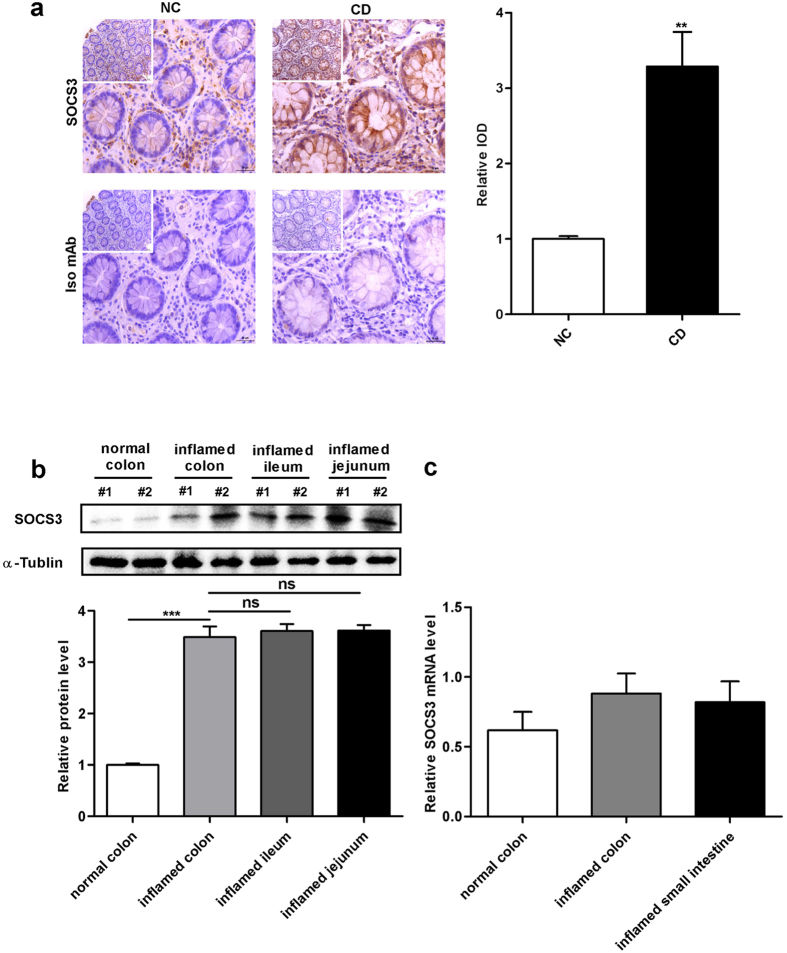
SOCS3 protein, but not SOCS3 mRNA, is upregulated in active CD intestinal mucosa tissue. **(a)**, Intestinal SOCS3 expression in normal control and CD patients was determined by immunohistochemical staining. No positive expression was observed in sites implanted with isotype-matched control Ab. The average IOD was obtained by analysing SOCS3 IHC in five random fields of each slide. Left panel: representative image; right panel: quantitative analysis. Results are presented as the mean ± SEM (low magnification, 200×, scale bars = 50 μm; high magnification, 400×, scale bars = 30 μm; n = 20 per group;^**^p = 0.001104). **(b)**, Western blot analysis of SOCS3 expression in the inflamed jejunum, ileum and colonic mucosa of CD patients and the colon of normal control patients. SOCS3 densitometry quantification. Relative expression was normalized to α-tubulin. Upper panel: representative image; lower panel: quantitative analysis from three independent experiments. The original whole-blot pictures are available in Supplementary Fig. S5. Results are presented as the mean±SEM (n = 20 per group; ^***^p = 0.0000, normal colon *vs.* CD inflamed colon; *p* = 0.277569, CD inflamed colon *vs.* CD inflamed ileum; *p* = 0.158925, CD inflamed colon *vs.* CD inflamed jejunum). **(c)**, Relative quantification of SOCS3 mRNA normalized to GAPDH in intestinal tissues from control and CD samples (n = 6; *p* = 0.107).

To evaluate SOCS3 expression and distribution in the intestinal mucosa, we performed quantitative RT-PCR, immunohistochemical (IHC) analysis and Western blot analysis. IHC analysis revealed that SOCS3 protein was highly expressed in the intestinal mucosa of active CD patients compared to normal controls, especially in the epithelial layer (Fig. 1a). In addition, the intensity of SOCS3 expression was significantly higher in sites with immobilized SOCS3 antibody, whereas expression was undetectable in sites labelled with isotype-matched control monoclonal antibody (mAb) (Fig. 1a). Based on Western blot analysis, SOCS3 protein was intensely expressed in the inflamed jejunum, ileum and colon mucosa of CD patients, whereas this expression was barely detectable in normal colon mucosa; there was no difference in SOCS3 expression between the jejunum, ileum and colon in CD patients (Fig. 1b). Interestingly, SOCS3 mRNA expression was not significantly different among inflamed colonic tissue and small intestinal mucosa from CD patients and normal intestinal mucosa from control subjects (Fig. 1c). This disparity between SOCS3 protein and mRNA expression in active CD suggests that SOCS3 expression is post-transcriptionally regulated.

### The SOCS3 3’-UTR contains conserved miR-19b target sites

**Figure 2:**
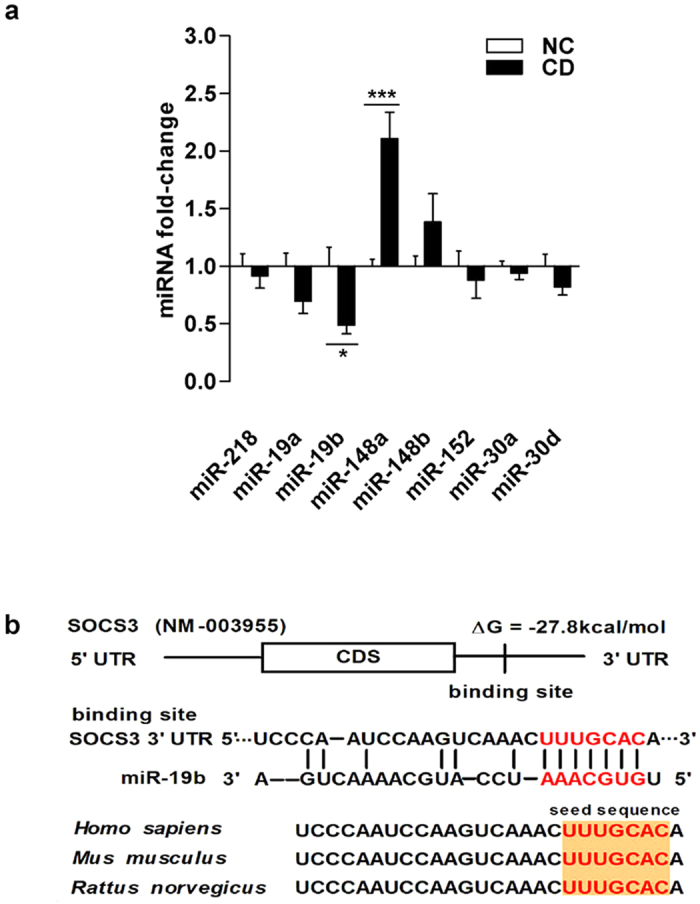
The SOCS3 3’-UTR contains conserved miR-19b target sites. **(a)**, The fold-changes of eight miRNAs predicted by three algorithms in CD intestinal tissues compared to controls, as analysed by qRT-PCR (n = 20 per group; ^*^p = 0.012, miR-19b group;^***^p = 0.000395, miR-148a group). **(b)**, Schematic illustration of conserved duplexes formed by SOCS3 3’UTR and miR-19b interactions. The predicted free energy of the hybrid is noted. The predicted structure of the base-paired hybrid is shown. Paired bases are marked by a black line. Sequence alignment of the putative miR-19b binding sites across species is shown. The complementary seed sites are marked in red, and all nucleotides in these regions are conserved among several species, including human, mouse and rat.

Because miRNA is an important post-transcriptional regulator of gene expression, we hypothesized that miRNAs regulate SOCS3 expression. We used the computer-aided algorithms TargetScan, PicTar and microRNA.org to predict miRNAs that could potentially target SOCS3. Using these approaches, we identified eight miRNAs (miR-218, miR-19a, miR-19b, miR-148a, miR-148b, miR-152, miR-30a, miR-30d) that could potentially bind to complementary sequences in the SOCS3 3’-UTR .We next examined the expression of these miRNAs in inflamed intestinal mucosa from active CD patients by TaqMan probe-based qRT-PCR. Among these miRNAs, miR-19b was downregulated and miR-148a was upregulated, whereas the expression levels of miR-218, miR-19a, miR-148b, miR-152, miR-30a, and miR-30d showed no changes in active CD (Fig. 2a). As shown in Figure 2b, we observed a miR-19b-SOCS3 3’-UTR hybrid; the minimum free energy value of miR-19b-SOCS3 hybridization was -27.8 kcal/mol, which is well within the range of genuinemiRNA-target pairs determined by RNAhybrid analysis^21^. Moreover, there was perfect base pairing between the seed region (the core sequence encompassing the first 2–8 bases of mature miRNA) and the cognate targets. Furthermore, miR-19b binding sequences in the SOCS3 3’-UTR were highly conserved across species (Fig. 2b). Thus, human SOCS3 is likely a direct miR-19b target.

### miR-19b and SOCS3 levels are inversely correlated in active CD intestinal tissues

**Figure 3:**
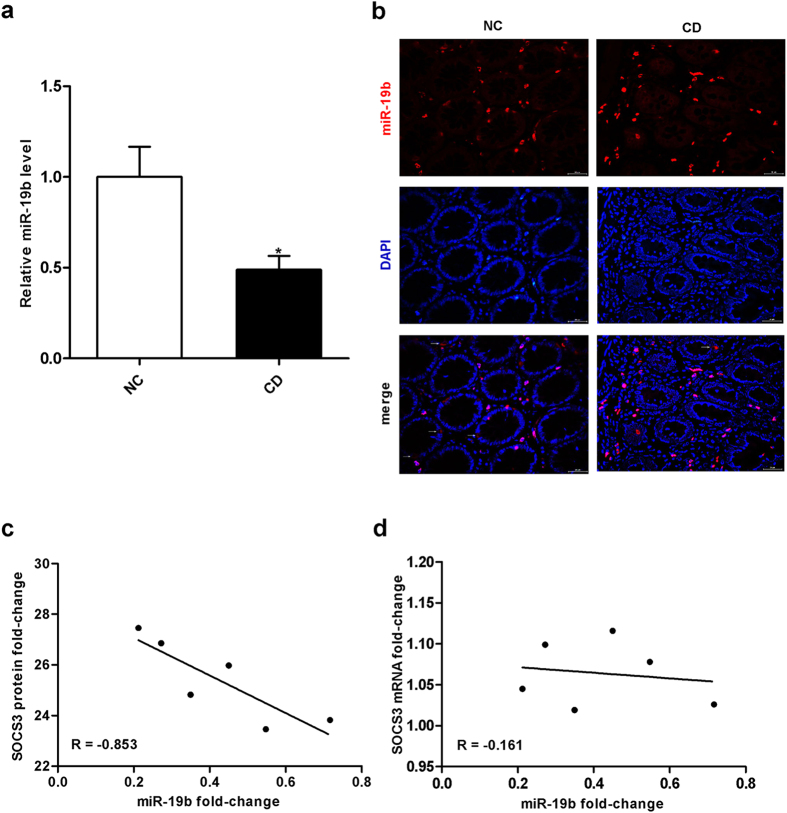
miR-19b and SOCS3 protein levels are inversely correlated in control and CD patient intestinal tissues. **** (**a**), Quantitative real-time PCR of miR-19b in control and CD patient intestinal tissues. The results are presented as the mean±SEM (n = 20 per group; ^*^p = 0.012). **(b)**, miRNA *in situ* hybridization for miR-19b was performed on intestinal sections from CD patients and normal controls (red, miR-19b; blue, DAPI nuclear staining). Magnification 400×, scale bar = 30 μm; n = 10 per group. **(c)**, Pearson’s correlation scatter plot of the miR-19b and SOCS3 protein expression fold-change in control and CD patient intestinal tissues (n = 6; ^*^p = 0.031). **(d)**, Pearson’s correlation scatter plot of miR-19b and SOCS3 mRNA fold-change in control and CD patient intestinal tissues (n = 6; *p* = 0.761).

miRNAs generally show opposite expression patterns when compared to their targets^22^. Thus, we investigated whether miR-19b was inversely correlated with SOCS3 in active CD. Using quantitative real-time PCR, we found that miR-19b expression was significantly decreased in inflamed intestinal mucosa from active CD patients compared to normal control samples (Fig. 3a). Using miRNA *in situ* hybridization, we detected miR-19b expression in epithelial cells and non-epithelial cells (Fig. 3b). In addition, miR-19b and SOCS3 protein expression were inversely correlated (Fig. 3c). The inverse correlation between miR-19b and SOCS3 protein level (Fig. 3c) and the disparity between miR-19b and SOCS3 mRNA levels (Fig. 3d) were further illustrated with Pearson’s correlation scatter plots. Animal miRNAs are thought to block translation without affecting transcript levels^22^. Our results indicate that SOCS3 expression is regulated by a typical miRNA-mediated, post-transcriptional mechanism. Based on computational predictions and the inverse correlation between miR-19b and SOCS3 protein levels in active CD, these data suggest that SOCS3 is a miR-19b target.

### Validation of human intestinal SOCS3 as a direct miR-19b target

**Figure 4:**
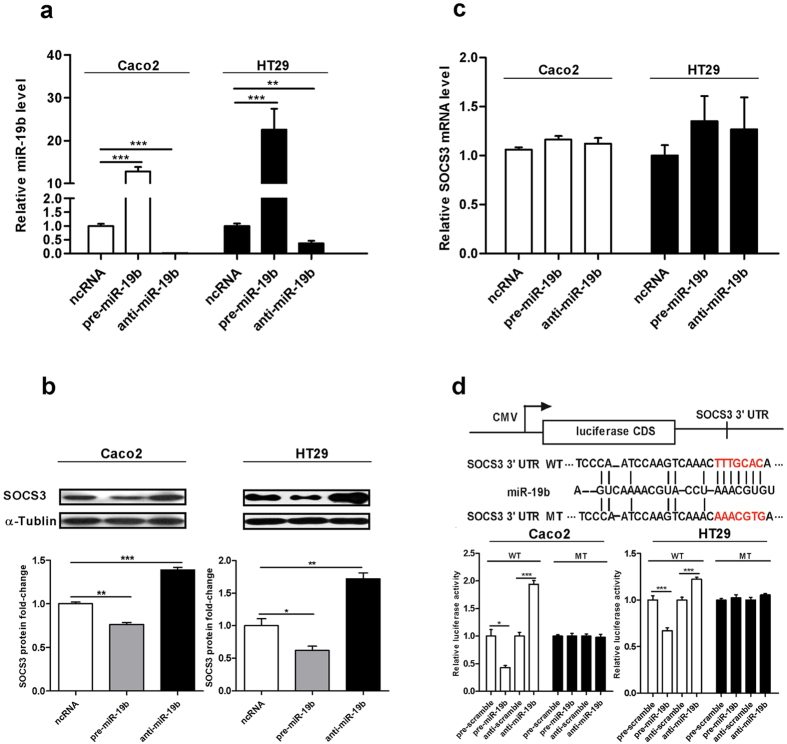
Validation of human SOCS3 as a direct miR-19b target.(**a**), Quantitative real-time PCR analysis of miR-19b expression in Caco2 cells and HT29 cells transfected with pre-miR-19b, anti-miR-19b or ncRNA. Left: Caco2 cells. Right: HT29 cells. Data are presented as the mean±SEM from three independent experiments (Caco2 group: ^***^p = 0.000092, ncRNA *vs.* pre-miR-19b, ^***^p = 0.000031, ncRNA *vs.* anti-miR-19b; HT29 group: ^***^p = 0.000253, ncRNA *vs.* pre-miR-19b,^**^p = 0.003, ncRNA *vs.* anti-miR-19b). (**b**), Immunoblot for endogenous SOCS3 in Caco2 cells and HT29 cells transfected with pre-miR-19b, anti-miR-19b or ncRNA. Left: Caco2 cells. Right: HT29 cells. α-Tubulin was used as a loading control. Top panel: representative image; Bottom panel: quantitative analysis. The original whole-blot pictures are available in Supplementary Fig. S6. Results are presented as the mean±SEM from three independent experiments (Caco2 group: ^**^p = 0.0014, ncRNA *vs.* pre-miR-19b,^***^p = 0.00036,ncRNA *vs.* anti-miR-19b; HT29 group: ^*^p = 0.038, ncRNA *vs.* pre-miR-19b,^**^p = 0.007, ncRNA *vs.* anti-miR-19b). **(c)**, Quantitative real-time PCR analysis of SOCS3 expression in Caco2 cells and HT29 cells treated with pre-miR-19b, anti-miR-19b or ncRNA. Left: Caco2 cells. Right: HT29 cells. The y-axis shows relative SOCS3 mRNA levels normalized to GAPDH. The results are presented as the mean±SEM from three independent experiments (*p* > 0.05 *vs.* ncRNA). **(d)**, Direct recognition of the SOCS3 3’-UTR by miR-19b. Firefly luciferase reporters containing wild-type (WT) or mutant (MT) miR-19b binding sites in the SOCS3 3’-UTR were co-transfected into Caco2 cells and HT29 cells with scrambled ncRNA, pre-miR-19b or anti-miR-19b. Twenty-four hours after transfection, a luciferase assay was performed. Firefly luciferase values were normalized to β-galactosidase activity and plotted as relative luciferase activity. The results were calculated as the ratio of firefly luciferase activity in miR-19b-transfected cells to the luciferase activity in control cells. Left: Caco2 cells. Right: HT29 cells. The results are presented as the mean±SEM from three independent experiments (Caco2 group: ^*^p = 0.013, *p*re-scramble *vs.* pre-miR-19b,^***^p = 0.0000, anti-scramble *vs.* anti-miR-19b; HT29 group:^***^p = 0.000021, pre-scramble *vs.* pre-miR-19b,^***^p = 0.000019, anti-scramble *vs.* anti-miR-19b).

We further examined the correlation between miR-19b and SOCS3 by altering the miR-19b level and examining the effect on SOCS3 expression in Caco2 cells and HT29 cells. We first overexpressed miR-19b by transfecting cells with pre-miR-19b, a synthetic RNA oligonucleotide duplex mimicking the miR-19b precursor. Then, we knocked down miR-19b by transfecting cells with anti-miR-19b, a chemically modified single-stranded antisense oligonucleotide designed to specifically target mature miR-19b. We validated these changes in miR-9b expression by quantitative RT-PCR 24 h after transfection (Fig. 4a).

To determine whether miR-19b overexpression or knockdown affected the SOCS3 level, we repeated the above experiments and examined SOCS3 expression 24 h after transfection. miR-19b overexpression significantly inhibited SOCS3 protein expression, whereas miR-19b knockdown significantly upregulated SOCS3 expression compared to the ncRNA control (Fig. 4b). In contrast, miR-19b overexpression or knockdown had no effect on SOCS3 mRNA stability (Fig. 4c). SOCS3 knockdown by RNA interference significantly decreased SOCS3 protein expression (Supplementary Fig. S1), while cells transfected with a SOCS3 overexpression plasmid expressed higher SOCS3 protein in both cell lines (Supplementary Fig. S1). These results demonstrate that miR-19b specifically regulates SOCS3 protein expression at the post-transcriptional level, which is a canonical mechanism of miRNA-mediated regulation.

To determine how miR-19b negatively affects SOCS3 expression, we fused full-length SOCS3 3’-UTR (1574bp) containing the presumed miR-19b binding site downstream of the firefly luciferase gene in a reporter plasmid. We co-transfected Caco2 cells and HT29 cells with the luciferase reporter and a transfection control β-galactosidase expression plasmid along with pre-miR-19b, anti-miR-19b or scrambled ncRNA. As expected, in both cell lines, miR-19b overexpression led to an approximate 50% reduction in luciferase reporter activity compared to control cells treated with scrambled ncRNA, whereas inhibition of miR-19b resulted in a significant increase in reporter activity compared to cells transfected with ncRNA (Fig. 4d). Furthermore, we introduced point mutations into the complementary sites of the SOCS3 3’-UTR to eliminate the predicted miR-19b binding site. As shown in Fig. 4d, the mutated luciferase reporter was not affected by altering the miR-19b level. These results suggest that the miRNA binding site strongly contributes to the miRNA-mRNA interaction that mediates post-transcriptional SOCS3 expression. Taken together, these results unequivocally demonstrate that miR-19 directly recognizes and binds the SOCS3 3’ UTR and inhibits its expression at the post-transcriptional level.

### miR-19b modulates chemokine production in Caco2 cells via SOCS3 downregulation

**Figure 5:**
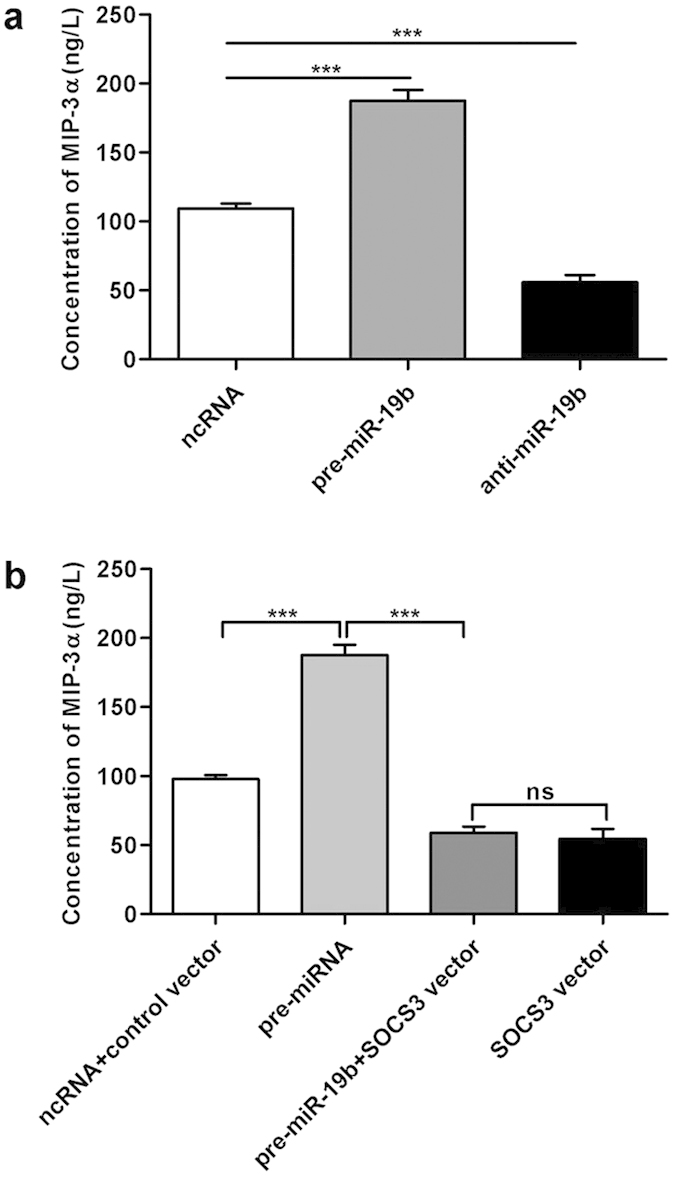
miR-19b modulates chemokine production in IECs via downregulation of SOCS3. **** (**a**), Caco2 cells were transfected with an equal dose of scrambled ncRNA, pre-miR-19b, or anti-miR-19b and then treated with IL-6 (100 ng/mL) for 24 h. MIP-3α expression was analysed by ELISA. The experiment was repeated three times, and the result is presented as the mean±SEM (^***^p = 0.000014, ncRNA *vs.* pre-miR-19b,^***^p = 0.000066, ncRNA *vs.* anti-miR-19b). (**b**), Caco2 cells transfected with pre-miR-19b and stimulated with IL-6 (100 ng/mL) for 24 h expressed higher levels of MIP-3α compared to cells treated with IL-6 alone; this outcome was reversed by SOCS3 overexpression. The results are presented as the mean±SEM from three independent experiments (^***^p = 0.000008, ncRNA+control vector *vs.* pre-miR-19b;^***^p = 0.000003, pre-miR-19b *vs.* pre-miR-19b+SOCS3 vector; ns, *p* = 0.153, *p*re-miR-19b+SOCS3 vector *vs.* SOCS3 vector).

To determine the function of miR-19b in IECs during intestinal inflammation, we evaluated the effects of miR-19b on chemokine production in IECs. First, chemokine levels were examined by chemokine array, and we observed alterations of chemokine production in Caco2 cells induced by IL-6 (shown in Supplementary Table S1, Table S2). We also found that the levels of IL-8 and CXCL16 were increased 72 h after IL-6 treatment. The level of macrophage-inflammatory protein-3α (MIP-3α) was increased 24 h after IL-6 treatment and remained high until 72 h after treatment. Therefore, we focused on these three chemokines (MIP-3α, IL-8 and CXCL16) in subsequent experiments. We first transfected Caco2 cells with pre-miR-19b or anti-miR-19b and then treated the cells with IL-6, and enzyme-linked immunosorbent assays (ELISAs) were performed to measure the levels of these three chemokines. As shown in Fig. 5a, MIP-3α was significantly increased upon miR-19b up-regulation and decreased upon miR-19b down-regulation 24 h after IL-6 treatment. However, the MIP-3α level was not significantly different 72 h after transfection with pre-miR-19b or anti-miR-19b (Supplementary Fig. S2). Similarly, the IL-8 and CXCL16 levels were not significantly different upon up-regulation or down-regulation of miR-19b after 72 h IL-6 treatment (Supplementary Fig. S2). We then transfected cells with siRNA to deplete SOCS3 and stimulated the cells with IL-6 for 24 h, and the results revealed increased levels of MIP-3α (Supplementary Fig. S3a). In contrast, transfection with the SOCS3 overexpression plasmid alone downregulated MIP-3α compared to control cells transfected with an empty plasmid control (Supplementary Fig. S3a). However, 72 h of IL-6 stimulation did not significantly affect the MIP-3α level regardless of the SOCS3 protein level (Supplementary Fig. S3b). We next investigated whether SOCS3 overexpression was sufficient to reverse the inhibitory effects of miR-19b on MIP-3α production in Caco2 cells. We co-transfected the SOCS3 overexpression plasmid and pre-miR-19b into Caco2 cells and examined the level of MIP-3α. Compared to cells transfected with pre-miR-19b, cells transfected with pre-miR-19b and the SOCS3 overexpression plasmid exhibited significantly lower production of MIP-3α, suggesting that miR-19b upregulation induced the increased expression of MIP-3α, which could be reversed by SOCS3 overexpression (Fig. 5b). Taken together, these results demonstrate that miR-19b promotes IEC chemokine production by down-regulating SOCS3 protein.

### miR-19b reduces intestinal inflammation *in vivo*

**Figure 6:**
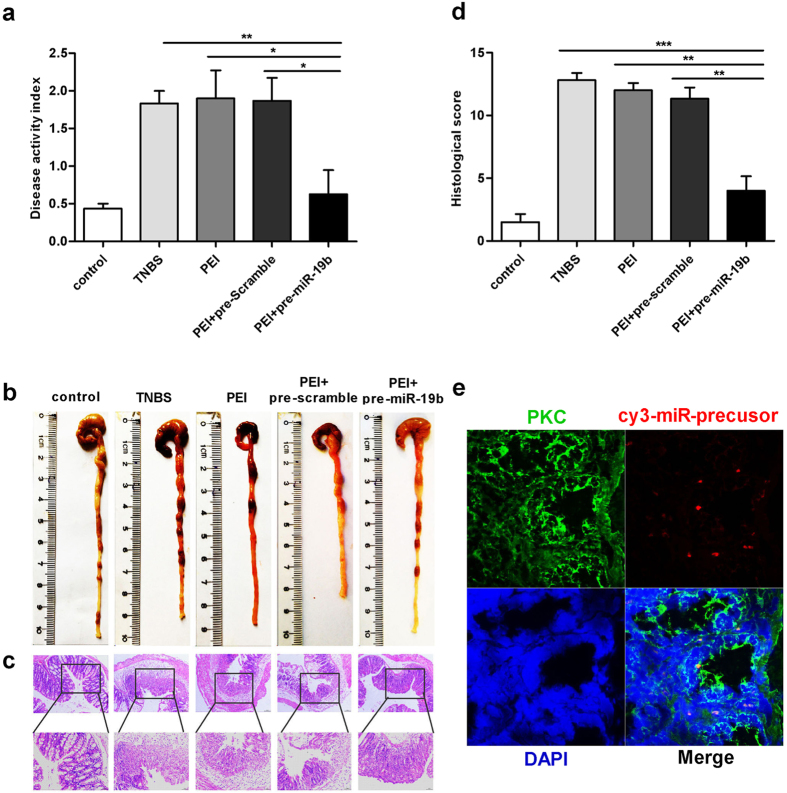
miR-19b reduces intestinal inflammation. **(a)**, DAI indices were monitored daily after pre-miR-19b treatment (n = 8 mice per group;^**^p = 0.006, TNBS group *vs.* PEI+pre-miR19b group; ^*^p = 0.024, PEI group *vs.* PEI+pre-miR-19b group;^*^p = 0.027, PEI+pre-scramble group *vs.* PEI+pre-miR-19b group). (**b**), Colons were removed for macroscopic observation three days after TNBS treatment. (**c**), Colon se**c**tions from control mice or TNBS**+**pre-miR-19b**-**treated mice were examined by H&E staining (Top panel: magnification, 100×, scale bars = 100 μm; Bottom panel: magnification, 200×, scale bars = 50 μm). (**d**), Histopathological scores were calculated according to H&E staining (n = 8 mice per group;^***^p = 0.0000256, TNBS group *vs.* PEI+pre-miR19b group; ^**^p = 0.003448, PEI group *vs.* PEI+pre-miR-19b group; ^**^p = 0.007246, PEI+pre-scramble group *vs.* PEI+pre-miR-19b group). (**e**), Immunofluoresence staining to determine miRNA precursor localization after intracolonic administration of the Cy3-labeled pre-miR-19b/PEI complex. Red, miR-19b-precusor; green, pan-cytokeratin (PCK); blue, DAPI nuclear staining (magnification, 400×). All values are expressed as the mean±SEM.

To investigate the potential anti-inflammatory effects of miR-19b on intestinal inflammation, we employed the 2,4,6-trinitrobenzene sulphonic acid (TNBS)-induced colitis model and treated these mice with pre-miR-19b, pre-scramble or 50% ethanol by intracolonic administration. As shown in Fig. 6a-d, the administration of TNBS to female BALB/c mice resulted in colonic damage, an increased disease activity index (DAI) (TNBS-induced mice: 1.8333 ± 0.16667 *vs.* control: 0.433 ± 0.06667) (Fig. 6a), and microscopic colonic damage (histology score: TNBS-induced mice: 12.8 ± 0.58310 *vs.* control: 1.5 ± 0.64550) (Fig. 6d). TNBS-induced mice recovered their body weight and showed a decreased DAI when treated with pre-miR-19b (TNBS-induced mice: 1.8333 ± 0.16667 *vs.* PEI+pre-miR-19b: 0.6250 ± 0.32185) (Fig. 6a). After pre-miR-19b treatment, TNBS-treated colons exhibited fewer signs of macroscopic inflammation, such as epithelial damage, oedema, inflammatory cell infiltration and necrosis, compared to mimic-control or ethanol-treated colons. Pre-miR-19b also prevented other inflammatory-associated changes, including decreases in colon length (Fig. 6b). We confirmed the anti-inflammatory effect of miR-19b at the histological level by haematoxylin and eosin (H&E) staining of colon sections (Fig. 6c). We found that TNBS-induced colitis affected all layers of the colon, causing submucosal oedema and strong leukocyte infiltration. However, treatment with pre-miR-19b induced a striking improvement in histological signs of inflammation, and the histological scores confirmed miR-19b’s anti-inflammatory effect (Fig. 6d). Furthermore, we detected Cy3-labeled pre-miR-19b in colon epithelial cells (Fig. 6e). Together, these results demonstrate that miR-19b decreases the severity of TNBS-induced colitis.

### The relationship between miR-19b and SOCS3 in the pathogenesis of colitis

**Figure 7:**
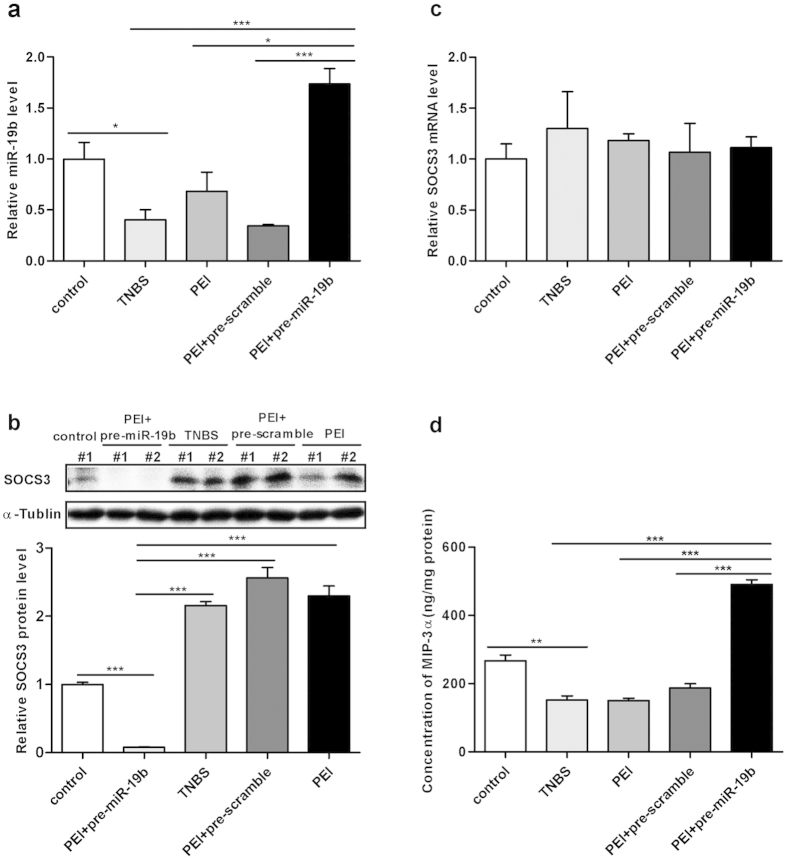
The relationship between miR-19b and SOCS3 in the pathogenesis of colitis**(a)**, Quantitative real-time PCR analysis of miR-19b levels in different treated groups. (n = 8 mice per group;^*^p = 0.021030, control group *vs*. TNBS group;^***^p = 0.000628, TNBS group *vs*. PEI+pre-miR19b group; ^*^p = 0.022823, PEI group *vs.* PEI+pre-miR-19b group;^***^p = 0.000837, PEI+pre-scramble group *vs.* PEI+pre-miR-19b group). **(b)**, The Western blot analysis of colon sections for different treated mice implanted with SOCS3 antibody is presented. Upper panel: representative image; lower panel: quantitative analysis from three independent experiments. The original whole blot pictures are available in Supplementary Fig. S7. (n = 8 mice per group;^***^p = 0.0000, normal control *vs*. PEI+pre-miR19b group;^***^p = 0.000015, TNBS group *vs.* PEI+pre-miR19b group;^***^p = 0.000010, PEI+pre-scramble group *vs*. PEI+pre-miR-19b group; ^***^p = 0.0000, PEI group *vs.* PEI+pre-miR-19b group). **(c)**, SOCS3 mRNA was normalized to GAPDH expression in colon tissues of treated mice by PCR. None significance were detected in all groups. **(d)**, Tissue homogenates from each group mice were subjected to ELISA to quantify MIP-3α protein levels. Results are expressed as ng of MIP-3α per mg of total homogenate protein (n = 8 mice per group;^**^p = 0.002473, control group *vs*. TNBS group;^***^p = 0.000077, TNBS group *vs.* PEI+pre-miR19b group;^***^p = 0.000015, PEI group *vs*. PEI+pre-miR-19b group;^***^p = 0.00013, PEI+pre-scramble group *vs*. PEI+pre-miR-19b group).

To investigate the relationship between miR-19b and SOCS3 in the pathogenesis of colitis, we employed the TNBS-induced colitis model and examined SOCS3 expression after intra-colonic pre-miR-19b, pre-scramble or 50% ethanol administration. As shown in Fig. 7a,b, the expression of miR-19b in TNBS-induced colitis was decreased, while the expression of SOCS3 in TNBS-induced colitis was increased compared with the control. However, SOCS3 protein in TNBS-induced colitis was decreased after intra-colonic administration with pre-miR-19b. When we analysed the alteration in SOCS3 mRNA before and after administration of pre-miR-19b, there was no significant alteration in SOCS3 mRNA after treatment with pre-miR-19b (Fig. 7c). Furthermore, the expression of MIP-3α in TNBS-induced colitis was increased after intra-colonic administration with pre-miR-19b (Fig. 7d). Together, these results suggest that miR-19b regulates SOCS3 protein expression and MIP-3α expression in TNBS-induced colitis.

## Discussion

The etiology and pathogenesis of IBD remains unclear, although evidence from animal models and humans has demonstrated that the abnormal function of IECs contributes to tissue damage and the intestinal inflammatory response^23^. The intestinal epithelium is considered a constitutive component of the mucosal immune system. In our study, we provide the first report of a direct and important role for miR-19b in regulating SOCS3 expression in CD-associated inflammation and highlight its potential as a miRNA-based therapeutic candidate. miRNAs are involved in the innate and adaptive immune systems, and significant efforts have been made to determine the expression of miRNAs in autoimmune disease and their roles in the pathogenesis, diagnosis, and treatment of several diseases, including CD^24^,^25^,^26^. However, previous CD studies have been limited by an incomplete understanding of the mechanisms regulating gene expression.

In this study, we identified a conserved miR-19b binding site in the human SOCS3 mRNA 3’-UTR. We examined miR-19b and SOCS3 expression levels in human inflammatory intestinal tissue and normal intestinal tissue and found an inverse correlation between the miR-19b level and SOCS3 protein level. We further demonstrated this correlation by luciferase reporter assay, which showed direct binding between miR-19b and the SOCS3 3’-UTR. We further confirmed that miR-19b directly inhibited SOCS3 post-transcriptional expression by over-expressing or knocking down miR-19b in Caco2 cells and HT29 cells. Our results showed a clear inverse relationship between the cellular miR-19b level and SOCS3 protein level in these two cell lines. We also demonstrated that miR-19b was expressed in both IECs and non-epithelial cells in intestinal tissue by miRNA *in situ* hybridization. Together, these results indicate that miR-19b is a negative regulator of SOCS3 expression in IECs.

SOCS3-related miRNAs (miR-30a, miR-30e and miR-19b) are upregulated in atherosclerotic tissues in the mouse arteriosclerotic vascular disease (ASVD) model^27^. Previous worked showed that miR-19b negatively regulates SOCS3 expression in atherosclerotic tissue, and our study demonstrated this phenomenon in inflammatory intestinal tissue.

Some studies have reported that SOCS3 expression is specifically increased in inflamed colonic areas in UC and CD patients. Indeed, SOCS3 contributes to enhanced IEC vulnerability during UC remission, suggesting that SOCS3 may be a useful therapeutic target for IBD clinical monitoring and early mucosal healing^28^. Our results also demonstrate that SOCS3 expression is upregulated in inflamed areas of active CD, which is consistent with previous reports.

Finally, we demonstrated that SOCS3 inhibition via miR-19b overexpression promoted MIP-3α production *in vitro*. Specifically, overexpression of miR-19b downregulated SOCS3 and consequently increased MIP-3α, whereas miR-19b knockdown induced SOCS3 expression and inhibited MIP-3α production. MIP-3α is a CC chemokine and is predominantly expressed by small intestine and colon epithelial cells^29^. MIP-3α plays an important homeostatic function in the intestine by regulating tissue turnover and epithelial maintenance^30^, and it is possible that MIP-3α also participates in colitis development.

Because our results established miR-19b as a suppressor of inflammation in active CD, we next investigated the possible therapeutic effect of miR-19b’s *in vivo*. We used polyetherimide to load pre-miR-19b into IECs. Compared to the control groups, pre-miR-19b treatment reduced colitis-associated weight loss and the DAI and improved histological signs of inflammation. We also found that miR-19b expression was decreased, while SOCS3 expression was increased, in TNBS-induced colitis, and this finding is similar to that seen in CD patients. Furthermore, the expression of SOCS3 was inhibited in TNBS-induced colitis model after treatment with pre-miR-19b. Together, these results demonstrate that miR-19b significantly decreased inflammation in TNBS-induced colitis, which may be due to miR-19b’s targeted regulation of SOCS3.

In summary, miR-19b downregulation and SOCS3 protein upregulation may serve as a molecular signature of CD. Specifically, our results show that miR-19b suppresses its target SOCS3 to regulate MIP-3α production and mediate intestinal epithelial homeostatic function, and these alterations may influence mucosal repair following intestinal inflammation.

It should be noted that miR-19b may have additional, non-epithelial effects. For example, the study by Liu *et al.* suggests that miR-17 and miR-19b are the two miRNAs in the miR-17-92 cluster responsible for promoting Th17 responses^31^. Our results from miRNA *in situ* hybridization show that miR-19b is expressed in both IECs and non-epithelial cells in intestinal tissue; thus, both epithelial and non-epithelial miR-19b-SOCS3 signals may be important for colitis development. However, the association between miR-19b and T cells requires further investigation.

## Materials and Methods

All methods were carried out in accordance with the approved guidelines.

### Human tissue

The Ethics Committee of Nanjing Medical University approved all study aspects. All patients and normal control individuals provided informed consent prior to the procedure. Intestinal tissue samples were obtained by pinch biopsy during endoscopic examination of active CD patients or normal control subjects undergoing screening colonoscopies at the First Affiliated Hospital of Nanjing Medical University (Nanjing, China). Tissue samples were immediately frozen in liquid nitrogen following pinch biopsies and stored at -80°C. The clinical features of the patients are listed in Table 1.

### Cell culture

Human intestinal enterocyte-like Caco2 cells and colonocyte-like HT29 cells were purchased from China Cell Culture Center (Shanghai, China). Caco2 cells were cultured in high-glucose (4.5 g/L) Dulbecco’s modified Eagle’s medium (DMEM; Gibco; Carlsbad, CA, USA) supplemented with 10% fetal bovine serum (FBS; Gibco) and 1% penicillin and streptomycin. HT29 cells were cultured in McCoy’s 5 A medium (Gibco) containing 1% penicillin and streptomycin and 10% FBS. All cells were incubated at 37 °C in a 5% CO_2_ humidified atmosphere.

### RNA isolation and quantitative RT-PCR

Total RNA was extracted from cultured cells and human intestinal tissues using TRIzol reagent (Invitrogen, Carlsbad, CA, USA) according to the manufacturer’s instructions. The total RNA concentration was measured with a BioPhotometer (Eppendorf, Hamburg, Germany). To quantify mature miRNA expression, TaqMan microRNA probes (Applied Biosystems, Foster City, CA, USA) were used according to the manufacturer’s instructions. Briefly, 1 μg of total RNA was reverse-transcribed into cDNA using Avian Myeloblastosis Virus (AMV) reverse transcriptase (TaKaRa, Dalian, China) and a stem-loop RT primer (Applied Biosystems, Foster City, CA, USA). The reaction conditions were as follows: 16 °C for 30 min, 42 °C for 30 min and 85 °C for 5 min. Real-time PCR was performed with a TaqMan PCR kit and an Applied Biosystems 7300 Sequence Detection System (Applied Biosystems). Reactions were incubated in a 96-well optical plate at 95 °C for 5 min, followed by 40 cycles at 95 °C for 15 s and 60 °C for 1 min. All reactions, including controls without template, were run in triplicate. Cycle threshold (CT) data were determined using fixed threshold settings, and the mean CT value was determined from triplicate PCRs. A comparative CT method was used to compare each condition to control reactions. U6 snRNA served as an internal control, and the relative amount of miRNA was normalized to U6 using the 2^-ΔΔCT^ equation, in which ΔΔCT = (C_T miR-19b_ - C_T U6_) _target_ - (C_T miR-19b_ - C_T U6_) _control._

To quantify SOCS3 and GAPDH mRNA expression, 1 μg total RNA was reverse-transcribed into cDNA using oligo(dT) 18 primers (TaKaRa) and ThermoScript reverse transcriptase (Invitrogen). The reaction conditions were as follows: 16 °C for 30 min, 42 °C for 30 min, and 85 °C for 5 min. SYBR Green dye (Invitrogen) was added to the reverse transcription (RT) product, and real-time PCR was performed.

PCR was performed in a 20 μL reaction containing 1 μL cDNA, 1X QuantiTect SYBR green PCR Master Mix (Applied Biosystems), and 0.5 μM sense and antisense primers. The primer sequences were as follows:

1. human SOCS3 (sense): 5′-GGAGTTCCTGGACCAGTACG-3′;
2. human SOCS3 (antisense): 5′-TTCTTGTGCTTGTGCCATGT-3′;
3. mouse SOCS3 (sense): 5′-ACCTTCAGCTCCAAAAGCGAGTAC-3′;
4. mouse SOCS3 (antisense): 5′-CGCTCCAGTAGAATCCGCTCTC-3′;
5. GAPDH (sense): 5′- AGAAGGCTGGGGCTCATTTG-3′;
6. GAPDH (antisense): 5′-AGGGGCCATCCACAGTCTTC-3′.

Reactions were incubated at 95 °C for 5 min, followed by 40 cycles of 95 °C for 15 s, 55 °C for 30 s, and 72 °C for 1 min. Following the reactions, CT values were determined by setting a fixed threshold. The relative amount of SOCS3 mRNA was normalized to that of GAPDH.

### Western blot analysis

Cold RIPA lysis buffer (Beyotime, China), supplemented with a protease and phosphatase inhibitor cocktail (Thermo Scientific, USA), was used to solubilize tissues and cells on ice for 30 min. Then, cell lysates or tissue homogenates were centrifuged for 15 min (12,000 *g*, 4 °C). The supernatant was collected, and the protein concentration was calculated with a BCA protein assay kit (Thermo Scientific, USA). A total of 60 μg protein from cell or tissue was separated by 10% SDS-PAGE and transferred to a polyvinylidene difluoride (PVDF) membrane (Bio-Rad Laboratories, USA), which was then blocked in 5% nonfat dried milk with PBS at room temperature for 2 h. The membrane was probed overnight at 4 °C with rabbit anti-SOCS3 antibody (1:1,000; Abcam, USA) or mouse anti-α-tubulin antibody (1:1,000, Santa Cruz Biotechnology, USA). Then, the membrane was incubated with goat-anti-rabbit or goat-anti-mouse secondary antibody, respectively (1:4,000, Bioworld Technology, China), for 60 min at room temperature. Enhanced chemiluminescence reagent was used to detected the signal on the membrane (Thermo Scientific, USA). Data were analysed by densitometry using ImageJ 1.42 software and normalized to α-tubulin expression. The blots shown in the figures were cropped for better presentation. The original whole-gel blots can be found in the supplementary data.

### Immunohistochemistry

Immunohistochemistry was performed on 5-μm sections of paraffin-embedded intestinal tissue specimens as described previously^32^. Briefly, the tissues were fixed in 4% paraformaldehyde and washed in 0.01 M PBS. Isotype-matched mAb (Iso, anti-rabbit IgG mAb, Equitech-Bio, USA) with no specific affinity to interest proteins was used as the negative control. Antibodies for SOCS3 (Abcam, USA), p-STAT3 (Cell Signaling Technology, USA) and STAT3 (Cell Signaling Technology, USA), as well as isotype control mAbs, were diluted in plain phosphate-buffered saline (PBS) at 25 μg/mL and immobilized on the tissue slides at 4 °C overnight, as previously described^33^. The average integrated optical density (IOD) of SOCS3 IHC staining in CD and normal control tissues was calculated with Image-Pro Plus software (version 5.0) as previously reported^34^. Five fields were analysed on each slide^35^.

### miRNA *in situ* hybridization

A 5’,3’-digoxin-conjugated miR-19b probe (Exiqon, Vedbaek, Denmark) was used for the miRNA *in situ* hybridization of miR-19b. Intestinal sections were first blocked with prehybridization buffer (3% BSA in 4 × saline-sodium citrate, SSC) for 20 min at 22-25 °C below the predicted probe melting temperature (Tm) in a humidified chamber, and then the miR-19b probe (10 ng/ml) in the hybridization buffer (10% dextran sulphate in 4×SSC) was hybridized with the tissue sections overnight at the same temperature. After washing the slides with washing buffer, the sections were stained with anti-digoxin rhodamine conjugate (1:100, Exon Biotech Inc, Guangzhou, China) at 37 °C for 1 h away from light. Then, the sections were stained with 4’,6-diamidino-2-phenylindole (DAPI) for nuclear staining. All fluorescence images were captured on a fluorescence microscope (Leica, Germany).

### miRNA target prediction

miRNA-SOCS3 pairs were selected using algorithms from TargetScan ( http://genes.mit.edu/targetscan/)^36^, PicTar ( http://pictar.mdc-berlin.de/)^37^, and microRNA.org ( http://www.microrna.org)^38^.

### Plasmid construction and luciferase reporter assay

A luciferase reporter assay was performed as previously described^39^ to test miR-19b binding to its target gene, human SOCS3. The entire human SOCS3 3’-UTR, containing a presumptive miR-19b complementary site (seed sequence, UUUGCAC), was PCR-amplified with the following primers:

sense: 5’-TGGGAGCTCAATGTCAGCCCAGTAAGTATTGGCCAGT-3’; antisense: 5’-GATAAGCTT-GTGCTCTTTATTATAAATTACTGAAATGTTTC-3’. Human genomic DNA was used as a template. PCR products were cloned into the pMIR-REPORT plasmid (Ambion), and insertion was confirmed by sequencing. To test binding specificity, the miR-19b seed sequence was mutated from UUUGCAC to AAACGTG. For luciferase reporter assays, 2 μg firefly luciferase reporter plasmid, 2 μg β-galactosidase (β-gal) expression vector (Ambion), and 100 pmol pre-miR-19b (GenePharma, Shanghai, China), anti-miR-19b (GenePharma), and scrambled ncRNA (GenePharma) were transfected into Caco2 cells or HT29 cells in 6-well plates using Lipofectamine 2000 reagent (Invitrogen) according to the manufacturer’s instructions. The β-gal vector was used as a control. Twenty-four hours after transfection, luciferase assays were performed (Promega, Madison, WI, USA). The reported data represent three independent experiments.

### miRNA overexpression and knockdown

miR-19b was overexpressed using pre-miR-19b, whereas knockdown was achieved with anti-miR-19b. Synthetic pre-miR-19b and anti-miR-19b and scrambled negative control RNA were purchased from GenePharma (Shanghai, China). Caco2 cells or HT29 cells were seeded in 6-well plates and transfected with Lipofectamine 2000 (Invitrogen) when the cells reached 70% confluence. In miR-19b overexpression experiments, 100 pmol of pre-miR-19b or ncRNA was added. For miR-19b knockdown, 100 pmol of anti-miR-19b was used. After 6 h, the medium was changed to DMEM or McCoy’s 5 A supplemented with 1% FBS. Cells were harvested 24 h or 48 h after transfection. The miR-19b expression level was analysed by quantitative RT-PCR, and the SOCS3 protein level was assessed by Western blot. Protein samples were normalized against α-tubulin.

### SOCS3 overexpression and knockdown

A mammalian expression plasmid containing the human SOCS3 open reading frame without a 3’-UTR was purchased from Invitrogen. An empty plasmid was used as a negative control. Depletion of SOCS3 activity was accomplished by transfecting Caco2 cells and HT29 cells with small interfering RNA (siRNA). siRNA sequences targeting SOCS3 were designed and purchased from GenePharma (Shanghai, China). A scrambled siRNA that did not target human SOCS3 was included as a negative control. The SOCS3 overexpression plasmid or siRNA was transfected into Caco2 cells or HT29 cells using Lipofectamine 2000 (Invitrogen) according to the manufacturer’s instructions.

### Cytokine analysis

Cytokine chips were used to detect cytokines in Caco2 cell culture supernatants according to the manufacturer’s protocol. MIP-3α in culture supernatants from human Caco2 cells using a commercial ELISA kit (Lianshuo Biological, Shanghai, China) according to the manufacturer’s instructions.

For the expression of MIP-3α in the TNBS-induced colitis model, full-thickness colonic tissue specimens of mice were homogenised in PBS containing a cocktail of protease inhibitors (KeyGEN Bio TECH, Nanjing, China) supplemented with 1 mM PMSF. After centrifugation at 12,000 g for 10 min at 4 °C, the total protein level of the supernatants was determined with the BCA protein assay (Thermo Scientific, Rockford, IL, USA). To measure MIP-3α, a mouse double-antibody sandwich ELISA kit (R&D Systems, USA) was developed as described^40^. The absorbance was measured at 450 nm and compared with the respective standard curve of the cytokines.

### Establishment of experimental colitis

All animal experiments were performed in accordance with the National Institute of Health’s Guide for the Care and Use of Laboratory Animals and were approved by the Animal Care and Use Committee of Nanjing Medical University (Nanjing, China).

Female BALB/c mice (6-8 weeks old) were obtained from the Laboratory Animal Center of Nanjing University (Nanjing, China). All animals received care according to Chinese legal requirements. TNBS-induced acute colitis in BALB/c mice was established as previously reported^41^. Briefly, 2.5 mg TNBS (Sigma-Aldrich, St. Louis, MO, USA) in 100 μL 50% ethanol was slowly administered into the colon lumen via a catheter inserted 4 cm into the colon through the anus. Mice were kept in a vertical position for 30 s. Control mice received 50% ethanol in saline alone. Mice were monitored daily for bleeding, body weight and stool consistency. Mice were sacrificed three days after colitis induction. Disease activity was evaluated according to the DAI^42^. Histological scores of H&E sections were graded from 0 to 4 according to previous reports^41^,^42^.

### Treatment

Polyetherimide (PEI, 25 kDa; Sigma)/pre-miRNA or pre-scramble complexes were prepared by mixing an aqueous PEI solution (4 mg/ml) with an equal volume of miRNA precursor or control solutions (2 mg/ml). A total volume of 100 μl of this solution was administered to mice via rectal enema. TNBS-treated mice were divided into three groups: PEI/pre-miR-19b treatment, PEI/pre-scramble treatment, and PEI alone. Twelve hours after TNBS injection, 5 mg pre-miRNA or pre-scramble/kg body weight was administered^43^. Twenty-four hours after intracolonic administration with the Cy3-labeled miRNA precursor (GenePharma, Shanghai, China)/PEI complex, frozen colon sections were collected for immunofluorescence staining to analyse miRNA precursor localization. Following treatment, colon lengths were measured, and histopathological analysis was performed blindly. The DAI was used to evaluate intestinal inflammation severity based on previously published literature^42^. The histological score was evaluated based on H&E staining, as previously described^41^,^42^. Additionally, intestinal tissues samples were evaluated by performing Western blot, mRNA expression analysis, ELISA and miRNA quantification, as described above.

### Statistical analysis

All images are representative of at least three independent experiments. Each qRT-PCR, luciferase reporter assay and cell viability assay was performed in triplicate, and each experiment was repeated several times. The results are presented as the mean ± SEM. *P-*values were evaluated with the two-tailed Student’s *t*-test using SPSS Statistics v20.0 (IBM), and *p* < 0.05 was considered significant.

## Author Contributions

X.C., X.Z., J.S. performed experiments, analyzed data, and wrote the manuscript. Y.Z., W.Z., J.Z. participated in part experiments. C.W., H.L., X.C. were partly involved in acquisition and analysis of data. R.S., K.Z. and C.Z. provided vital guidance to the study. H.Z. conceived the study, designed and supervised the experiments, analyzed data, and wrote the manuscript. All authors read and approved the final manuscript.

## Additional Information

**How to cite this article**: Cheng, X. *et al.* miR-19b downregulates intestinal SOCS3 to reduce intestinal inflammation in Crohn's disease. *Sci. Rep.* **5**, 10397; doi: 10.1038/srep10397 (2015).

## Supplementary Material

Supplementary Information

## Figures and Tables

**Table 1:** Patients’ clinical features.

**Characteristics**	**Normal Control**	**Crohn’s Disease**
	**(n=20)**	**(n=20)**
Gender		
male	11	12
female	9	8
Mean age, years±SD	36.2±10.0	29.2±10.5
Mean duration of disease, years±SD	–	16.3±12.5
		
Diseased intestinal regions		
ileum	–	7
ileocolon	–	9
colon	–	4
Treatment		
prednisone	–	8
5-AZA+prednisone	–	7
IFX+prednisone	–	3
IFX+AZA	–	2
