# Supplementary material for: miR-19b downregulates intestinal SOCS3 to reduce intestinal inflammation in Crohn’s disease
Source: Sci Rep. 2015 May 22;5:10397. doi: 10.1038/srep10397 (PMC4441154; doi:10.1038/srep10397)
Supplement: Supplementary Information [file srep10397-s1.doc]

**miR-19b downregulates intestinal SOCS3 to reduce intestinal inflammation in Crohn’s disease**

Xiuqin Cheng^1#^, Xiaofei Zhang^1,3#^, Jiewen Su^1#^, Yingdi Zhang^1^, Weimei Zhou^1^, Jun Zhou^1^, Cheng Wang^2^, Hongwei Liang^2^, Xi Chen^2^, Ruihua Shi^1^, Ke Zen^2^, Chen-Yu Zhang^2^, Hongjie Zhang^1*^

**Figure S1. Western blot for SOCS3 protein in Caco2 cells and HT29 cells transfected with SOCS3 siRNA or SOCS3 overexpression vector**

**a:** Western blot for SOCS3 protein in Caco2 cells. **b:** Western blot for SOCS3 protein in HT29 cells. Left: SOCS3 siRNA transfection, Right: SOCS3 overexpression vector transfection. α-Tubulin was used as a loading control. Top panel: representative image; Bottom panel: quantitative analysis. The results are presented as the mean±SEM from three independent experiments (a: **^***^***p*=0.00489, control siRNA *vs.* SOCS3 siRNA; **^***^***p*=0.000068, control vector *vs.* SOCS3 vector; b: **^**^***p*=0.003, control siRNA *vs.* SOCS3 siRNA; **^***^***p*=0.000234, control vector *vs.* SOCS3 vector).

**Figure S2. MIP-3α, IL-8 and CXCL16 expression levels in Caco2 cells after transfection with pre-miR-19b or anti-miR-19b**

Caco2 cells were transfected with pre-miR-19b, anti-miR-19b or ncRNA and treated with IL-6 (100 ng/mL) for 72 h. MIP-3α, IL-8 and CXCL16 expression levels were analyzed by ELISA. The experiment was repeated three times, and results are presented as the mean±SEM (p>0.05 *vs*. ncRNA).

**Figure S3.** **The effect of SOCS3 knockdown or overexpression on MIP-3α production in Caco2 cells 24 h or 72 h after IL-6 treatment**

MIP-3α concentration in supernatants from Caco2 cells transfected with SOCS3 siRNA or SOCS3 overexpression vector were verified 24h(Fig. S3a）and 72h (Fig. S3b) after IL-6 treatment (100 ng/mL) by ELISA. Left: MIP-3α level in culture supernatants from cells transfected with control siRNA or SOCS3 siRNA. Right: MIP-3α level in culture supernatants from cells transfected with control vector or SOCS3 overexpression. The results are presented as the mean±SEM from three independent experiments (**^***^***p=*0.000259, control siRNA *vs.* SOCS3 siRNA; **^**^***p*=0.002, control vector *vs*. SOCS3 vector).

**a**

**b**

**Figure S4.** **Down-regulation of intestinal p-STAT3 but not STAT3 expression in CD patients than normal control detected by immunohistochemical staining**

Intestinal p-STAT3 and STAT3 expression in normal control and CD patients was determined by immunohistochemical staining. IHC analysis revealed that p-STAT3 protein in intestinal mucosa of active CD was little expressed while intensely detected in normal control(Fig. S4a).Both in normal control and active CD patients,STAT3 was highly expressed in the mucosa and there was no difference between these two groups(Fig. S4b). No positive expression was observed in sites implanted with isotype-matched control Ab. The average integrated optical density (IOD) was obtained by analyzing protein IHC in five random fields of each slide. Left panel: representative image; right panel: quantitative analysis. Results are presented as mean±SEM (low magnification, 200×, scale bars = 50μm, high magnification, 400×, scale bars = 30μm, n=10 per group; **^**^***p*=0.0065).

**a**

**NC CD**


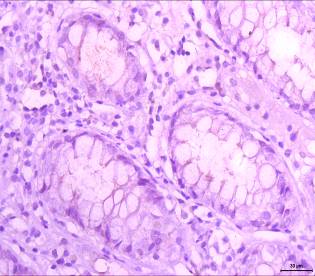

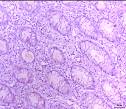

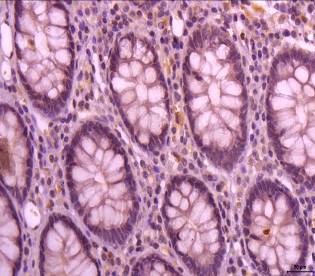

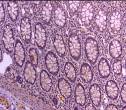


**p-STAT3**


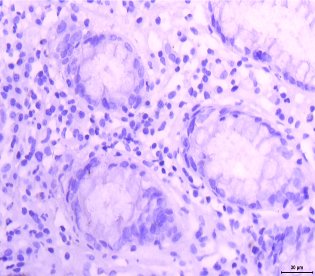

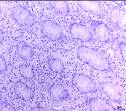

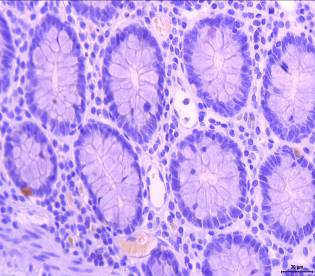

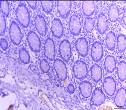


**Iso mAb**

**b**


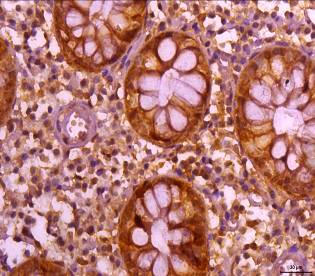

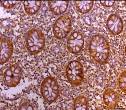

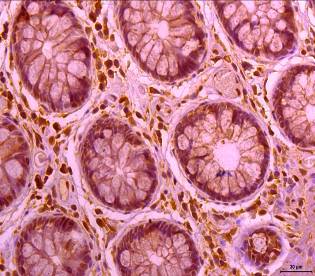

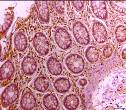

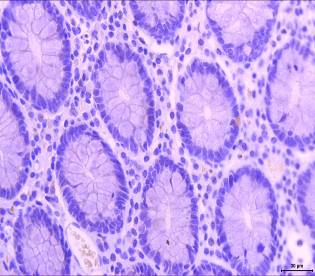

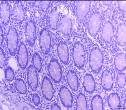


**STAT3**


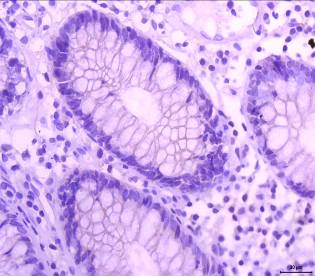

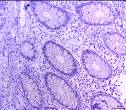


**Iso mAb**

**Figure S5. Full length gel images for the western blot analysis of Figure 1b.**

**
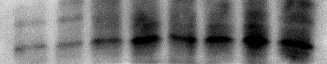
**

SOCS3

30kDa

**
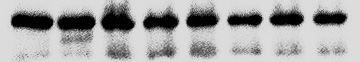
**

α-Tublin

55kDa

#1 #2 #1 #2 #1 #2 #1 #2

inflamed

ileum

inflamed

jejunum

normal

colon

inflamed

colon

**Figure S6. Full length gel images for the western blot analysis of Figure 4b.**

HT-29

**
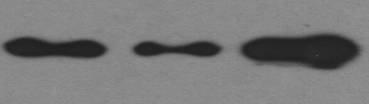
**

SOCS3

30kDa

**
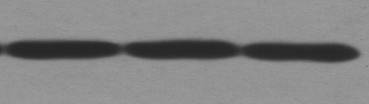
**

α-Tublin

55kDa

ncRNA

pre-

miR-19b

colon

anti-

miR-19b

**Figure S7.** **Full length gel images for the western blot analysis of Figure 7b.**


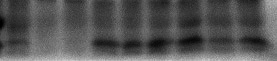


SOCS3

30kDa

55kDa

**
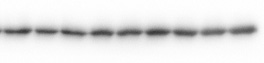
**

α-Tublin

#1 #1 #2 #1 #2 #1 #2 #1 #2

a b c d e

a:control b:PEI+pre-miR-19b c:TNBS d:PEI+pre-scramble e:PEI

**Figure S8.** **Full length gel images for the western blot analysis of Figure S1b.**

**
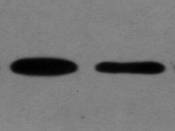

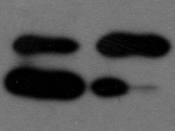
**

SOCS3

30kDa

**
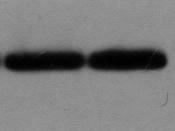

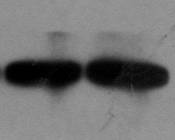
**

α-Tublin

55kDa

control

siRNA

SOCS3

siRNA

control

vector

SOCS3

vector

**Table S1. Cytokine concentrations in Caco2 cell supernatants were detected by chemokine arrays 24 h after IL-6 treatment (100 ng/mL). Fold-change was normalized to IL-6-untreated control cells.**

| **Analyte** | **Alternate**  **Nomenclature** | **Untreated** | **IL-6 Treated** | **Fold**  **Change** |
| --- | --- | --- | --- | --- |
|  |  | **(Mean Pixel Density)** | **(Mean Pixel Density)** |  |
| **MIP-3ɑ** | **CCL20,LARC,**  **Exodus-1** | **1314.03** | **6595.86** | **5.02** |
| **CXCL16** | **SRPSOX** | **4016.50** | **4600.03** | **1.15** |
| **IL-8** | **CXCL8** | **3285.74** | **4666.15** | **1.42** |
| **GRO-ɑ** | **CXCL1** | **14627.15** | **15751.03** | **1.08** |
| **Midkine** |  | **12743.62** | **15393.86** | **1.21** |

**Table S2. Cytokine concentrations in Caco2 cell supernatants were detected by chemokine arrays 72 h after IL-6 treatment (100 ng/mL). Fold-change was normalized to IL-6-untreated control cells.**

| **Analyte** | **Alternate**  **Nomenclature** | **Untreated** | **IL-6 Treated** | **Fold**  **Change** |
| --- | --- | --- | --- | --- |
|  |  | **(Mean Pixel Density)** | **(Mean Pixel Density)** |  |
| **MIP-3ɑ** | **CCL20,LARC,**  **Exodus-1** | **19742.89** | **37981.69** | **1.92** |
| **CXCL16** | **SRPSOX** | **307.26** | **13137.11** | **42.76** |
| **IL-8** | **CXCL8** | **4524.88** | **18078.77** | **4.00** |
| **ENA-78** | **CXCL5** | **662.19** | **1479.83** | **2.23** |
| **Fractalkine** | **CX3CL1,**  **Neurotactin** | **3846.99** | **3470.84** | **0.90** |
| **GRO-ɑ** | **CXCL1** | **34312.48** | **43962.23** | **1.28** |
| **IP-10** | **CXCL10** | **1032.55** | **3072.83** | **2.98** |
| **Midkine** |  | **32809.70** | **37845.89** | **1.15** |
| **MIP-1δ** | **CCL15，MIP-5,**  **Leukotactin1,**  **HCC-2** | **3098.90** | **3080.73** | **0.99** |
| **NAP-2** | **CXCL7,CTAPIII** | **1984.65** | **3789.44** | **1.91** |
| **TARC** | **CCL17** | **1932.11** | **2787.55** | **1.44** |
